# Targeted treatment in a case series of AR+, HRAS/PIK3CA co-mutated salivary duct carcinoma

**DOI:** 10.3389/fonc.2023.1107134

**Published:** 2023-06-20

**Authors:** Damian T. Rieke, Sebastian Schröder, Philippe Schafhausen, Eric Blanc, Erika Zuljan, Benjamin von der Emde, Dieter Beule, Ulrich Keller, Ulrich Keilholz, Konrad Klinghammer

**Affiliations:** ^1^ Department of Hematology, Oncology and Cancer Immunology, Charité – Universitätsmedizin Berlin, Corporate Member of Freie Universität Berlin and Humboldt-Universität zu Berlin, Berlin, Germany; ^2^ Comprehensive Cancer Center, Charité – Universitätsmedizin Berlin, Corporate Member of Freie Universität Berlin and Humboldt-Universität zu Berlin, Berlin, Germany; ^3^ Berlin Institute of Health (BIH) at Charité – Universitätsmedizin Berlin, Berlin, Germany; ^4^ German Cancer Consortium (DKTK) and German Cancer Research Center (DKFZ), Heidelberg, Germany; ^5^ Department of Oncology, Hematology, and Bone Marrow Transplantation with Section of Pneumology, University Medical Center Hamburg-Eppendorf, Hamburg, Germany; ^6^ Core Unit Bioinformatics (CUBI), Berlin Institute of Health at Charité – Universitätsmedizin Berlin, Berlin, Germany; ^7^ Max-Delbrück-Center for Molecular Medicine, Berlin, Germany

**Keywords:** salivary gland cancer, salivary duct carcinoma, targeted therapy, precision oncology, molecular tumor board, head and neck cancer

## Abstract

**Background and purpose:**

A subgroup of salivary duct carcinoma (SDC) harbor overexpression of the androgen receptor (AR), and co-occurring mutations in the *HRAS*- and *PIK3CA*-genes. The impact of genomic complexity on targeted treatment strategies in advanced cancer is unknown.

**Materials and methods:**

We analyzed molecular and clinical data from an institutional molecular tumor board (MTB) to identify AR+, *HRAS*/*PIK3CA* co-mutated SDC. Follow-up was performed within the MTB registrational study or retrospective chart review after approval by the local ethics committee. Response was assessed by the investigator. A systematic literature search was performed in MEDLINE to identify additional clinically annotated cases.

**Results:**

4 patients with AR+ *HRAS*/*PIK3CA* co-mutated SDC and clinical follow-up data were identified from the MTB. An additional 9 patients with clinical follow-up were identified from the literature. In addition to AR overexpression and *HRAS* and *PIK3CA*-alterations, PD-L1 expression and Tumor Mutational Burden > 10 Mutations per Megabase were identified as additional potentially targetable alterations. Among evaluable patients, androgen deprivation therapy (ADT) was initiated in 7 patients (1 Partial Response (PR), 2 Stable Disease (SD), 3 Progressive Disease (PD), 2 not evaluable), tipifarnib was initiated in 6 patients (1 PR, 4 SD, 1 PD). One patient each was treated with immune checkpoint inhibition (Mixed Response) and combination therapies of tipifarnib and ADT (SD) and alpelisib and ADT (PR).

**Conclusion:**

Available data further support comprehensive molecular profiling of SDC. Combination therapies, PI3K-inhibitors and immune therapy warrant further investigation, ideally in clinical trials. Future research should consider this rare subgroup of SDC.

## 1 Introduction

Salivary gland cancers (SGC) are a rare group of tumors with an incidence of about 1.3 cases/100,000 individuals in the United States (1). More than 20 distinct malignant subtypes have been described, many of which are defined by recurrent genetic alterations (2).

Salivary duct carcinoma (SDC) is an aggressive high-grade SGC subtype with a dismal prognosis. SDC most commonly arises in the parotid gland and accounts for about 1.8% of major salivary gland tumors in the SEER database (3–5). SDC can arise *de-novo* or ex pleomorphic adenoma (ex-PA) (4). Due to the aggressive nature of this disease, metastatic spread and a need for systemic therapy is frequent (6).

In addition to chemotherapy, targeted treatment strategies are increasingly used in SDC. SDC harbors recurrent molecular alterations such as HER2 and androgen receptor (AR) amplification and overexpression. Furthermore, *FGFR1* amplification, *PIK3CA*, *HRAS* and *TP53* mutations and *PTEN* and *CDKN2A* loss have been described (2, 4, 7, 8). Some of these alterations have been applied as predictive biomarkers for targeted therapy. Previous prospective studies have shown activity of HER2, AR and *HRAS*-directed therapy in SDC (9–11). Additionally, a prospective basket study showed a benefit of targeted therapy (targeting *HER2* amplification, *HER2*, *BRAF* and *PTCH1* mutation and high tumor mutational burden) in a large group of SGC, including SDC (12). No prospective trials supporting the efficacy of PI3K-inhibitors in *PIK3CA*-mutant SDC currently exist. A summary of ongoing and published clinical trials relevant for metastatic salivary duct carcinoma is provided in **Table 1**.

**Table 1:** Published and ongoing clinical trials relevant for salivary duct carcinoma, as identified from a structured search (MEDLINE clinical trials, search term “salivary gland cancer” on 17^th^ May 2023, clinicaltrials.gov, search term “salivary gland AND metastatic” on 17^th^ May 2023).

Studyname (Identifier)	Recruitment status	Intervention	Phase	Reference
EORTC 1206 Androgen Deprivation Therapy in Advanced Salivary Gland Cancer	completed	bicalutamide + triptorelin	II, randomized	NCT01969578
Testing the Anti-Cancer Drug Darolutamide in Patients With Testosterone-driven Salivary Gland Cancers	recruiting	darolutamide	II, nonrandomized	NCT05669664
Abiraterone Acetate in Patients With Castration-Resistant, Androgen Receptor-Expressing Salivary Gland Cancer: A Phase II Trial	completed	abiraterone	II, nonrandomized	(13)
Phase II Study of Enzalutamide for Patients With Androgen Receptor-Positive Salivary Gland Cancers (Alliance A091404)	completed	enzalutamide	II, nonrandomized	(14)
Tipifarnib in recurrent, metastatic HRAS-mutant salivary gland cancer	completed	tipifarnib	II, nonrandomized	(9)
A prospective phase II study of combined androgen blockade in patients with androgen receptor-positive metastatic or locally advanced unresectable salivary gland carcinoma	completed	leuprorelin + bicalutamid	II, nonrandomized	(10)

These results have led to a recommendation of comprehensive molecular analyses (e.g. next-generation panel or whole-exome sequencing) in patients with advanced SDC. These analyses should be done to assess opportunities for targeted therapy, including HER2- or AR-directed treatment (15, 16). Available data correspond to ESMO Scale of Clinical Actionability (ESCAT) scores of II-B (i.e. investigational therapy, alteration-drug match is associated with antitumor activity but magnitude of benefit is unknown) for AR (>70% positivity by immunohistochemistry, IHC) and HER2 (IHC score 3+ or fluorescence *in situ* hybridization positivity) in SGC. A participation in clinical trials is strongly recommended (15–17). The use of immune checkpoint inhibition remains investigational (15, 16). However, the FDA-approval of pembrolizumab in tumors with high tumor mutational burden also includes SGC (18).

In SDC, several targetable molecular alterations occur in recurrent patterns. The resulting subgroups of SDC are mainly defined by HER2- and AR-expression. In a retrospective analysis of 63 SDC samples, 34 samples were AR+/HER2- and harbored frequent *PIK3CA* (50%) and *HRAS (41%)* mutations (19). In this study, *HRAS*-mutations were exclusively found in the HER2-/AR+ group and in 93% of cases they co-occurred with a *PIK3CA*-mutation (19). Additionally, no *HRAS* mutations were identified in SDC ex pleomorphic adenoma (19). The co-occurrence of three potentially predictive biomarkers complicates selection for targeted treatment decisions in these rare patients. We here present a case series of patients presenting to an institutional molecular tumor board or identified through a systematic search of the literature to assess outcome of AR+, *HRAS*/*PIK3CA* SDC patients with targeted treatment.

## 2 Materials and methods

### 2.1 Patients

Patients with salivary gland cancer presenting to the molecular tumor board (MTB) of the Charité Comprehensive Cancer Center between 2016 and 2022 were analyzed in a retrospective analysis of the MTB database (20). Original histopathological reports for patients classified as adenocarcinoma NOS, carcinoma NOS, invasive ductal carcinoma or carcinoma ex pleomorphic adenoma were considered. Patients with a final histopathological diagnosis of salivary duct carcinoma and molecular results with AR positivity (any immunohistochemistry, IHC staining) and activating *HRAS* and *PIK3CA*-mutations were included in the analysis (**Supplementary Figure 1**). Next-generation sequencing (NGS) was performed on formalin-fixed, paraffine-embedded tumor tissue for all identified patients, using the SureSelect Custom Library Panel (MH IVD Panel 600+, Agilent Technologies, USA). Library preparation was done using the SureSelectXT Low Input Target Enrichment System (Agilent Technologies, USA). Sequencing was performed on the NextSeq550 system using the NextSeq 500/550 Mid Output Kit v2.5, 300 Cycles (Illumina, USA). Follow-up, including response assessment, progression-free survival (PFS) and overall survival (OS), was performed prospectively within the MTB registrational study or as retrospective chart review. Median follow-up was calculated from the time of diagnosis. No minimum follow-up was required. The analysis was approved by the local ethics committee (Berlin, EA1/305/21).

### 2.2 Literature search

Systematic literature search (performed by DTR, last updated on 7^th^ November 2022) was performed on MEDLINE using the following terms: “PIK3CA AND HRAS AND SALIVARY” OR “AR AND SALIVARY DUCT CARCINOMA”. Studies and case reports providing individual clinical follow-up data for patients with AR+, *HRAS*/*PIK3CA* co-mutated cases were included in the analysis.

### 2.3 Analysis

Clinical patient characteristics, line and type of treatment, best response, time on treatment, progression-free survival and overall survival were collected, as provided. Best response was assessed by the investigator after a review of CT or MRI radiology reports (complete response, CR; partial response PR; stable disease SD; mixed response, MR; progressive disease, PD). Clinical benefit was defined as CR/PR or SD lasting for at least 6 months. Outcomes with similar treatment strategies (e.g. chemotherapy, androgen deprivation therapy, HER2-directed therapy, HRAS-directed therapy, combination therapy or immune checkpoint inhibition) were summarized. No formal statistical analysis was performed because of insufficient sample size. Cases were consecutively numbered starting with cases retrieved from the internal MTB database and followed by cases identified from the literature.

## 3 Results

### 3.1 Patient cohort

Seventeen patients with salivary gland histologies, consistent with SDC, were discussed in the institutional molecular tumor board between 2016 and 2022. After review of final histopathological diagnoses, 4 patients had salivary duct carcinoma with AR expression and *HRAS*/*PIK3CA* mutation and were included in the analysis. These patients (3 male, 1 female) were between 48-79 years old at the time of presentation at the MTB. Activating *HRAS* mutations were identified in the p.Q61 (3 patients) and p.G13 (1 patient) positions. Activating *PIK3CA* mutations were identified in the p.H1047 (3 patients) and p.E545 (1 patients) positions. Additional molecular findings were low to medium HER2-expression in 3 patients, PD-L1 expression in 2 patients, a tumor mutational burden (TMB) > 10 mutations/Megabase (mut/Mb) and an *AR* mutation in 1 patient, each. Median follow-up was 14.5 months. Clinical and molecular findings were summarized in **Table 2**.

**Table 2:** Clinical and molecular data for patients identified from the local MTB database.

ID	Age	Gender	Primary Site	Stage at diagnosis	Sites of metastases	Site sequenced	AR (IHC)	HER2 (IHC)	*HRAS* mutation	*PIK3CA mutation*	TMB (Mut/Mb)	Other Alterations
1	48	m	Parotid Gland	pT3pN2bcM1	lung	primary	positive	negative	p.G13V	p.E545K	2.9	*CDKN2A* mutation, PD-L1 CPS 45, *ARID1A* mutation, *TP53* mutation
2	61	f	Submandibular Gland	cT4cN3bcM0	skin	Skin metastasis	80%	2+	p.Q61R	p.H1047L	3.64	PD-L1 CPS 5, *AR* p.R20P
3	54	m	Parotid Gland	pT3N0M0	bone, lung	Bone metastasis	90%	1+	p.Q61K	p.H1047R	2.18	
4	79	m	Parotid Gland	cT4cN1cM1	lung	Lung metastasis	strong positive	1+	p.Q61R	p.H1047R	10.9	*NQO1* mutation

CPS, combined positivity score, f, female, ID, identification number; IHC, immunohistochemistry, m, male, Mut/Mb, mutations per megabase, TMB, tumor mutational burden.

The medline searches revealed 37 and 89 results, respectively. Of these, 4 studies with individual follow-up data for patients with AR+, *PIK3CA*/*HRAS* co-mutated SDC were included after manual review of the identified publications. The publications yielded a total of 9 cases (7 male, 2 female). Age was reported for 5 patients (range 38-65 years). Concurrent molecular alterations were *HER2* amplification and overexpression in 1 and *TP53* mutations in 2 patients, respectively. Clinical and molecular findings in these patients were summarized in **Table 3**. A consort diagram of patient identification is provided in **Supplementary Figure 1**.

**Table 3:** Clinical and molecular data for patients identified from literature review.Two PIK3CA mutations were identified in patient 12.

ID	Reference	Age	Gender	AR (IHC)	HER2 (IHC)	*HRAS* mutation	*PIK3CA* mutation	Other Alterations	Sequencing technique
5	(21)	64	m	positive	3+	p.Q61R	p.E545K	*HER2* amplification, *TP53* p.R196*, *ACVR2A* p.D177E	NGS
6	(9)	61	m	positive		p.Q61R	mutation	*TP53* mutation	NGS
7	(9)	65	m	n/a		p.Q61R	mutation		NGS
8	(9)	38	m	positive	2+	mutation	mutation		NGS
9	(22)	61	m	positive		p.Q61K	p.E545K		unknown
10	(23)	n/a	f	negative, mRNA pathway score 43.7	positive	p.Q61K	p.H1047R		NGS
11	(23)	n/a	f	positive		p.Q61R	p.E545K		NGS
12	(23)	n/a	m	positive		p.Q61K	p.E545K, p.H1047R		NGS
13	(23)	n/a	m	positive		p.Q61R	p.H1047R		NGS

AR IHC results are provided as described in the respective publications. No AR IHC was provided for patients #7. Results from a qPCR-based AR mRNA pathway activity test (normalized score, 0 lowest, 100 highest) was provided in patient #10.

ID, identification number; IHC, immunohistochemistry; n/a, not available; NGS, next generation sequencing.

Overall, 13 patients (10 male, 3 female; median age in 9 evaluable patients 61 years, range 38-79 years) with AR+, *PIK3CA*/*HRAS* co-mutated SDC were identified.

### 3.2 Treatment

**Figure 1:**
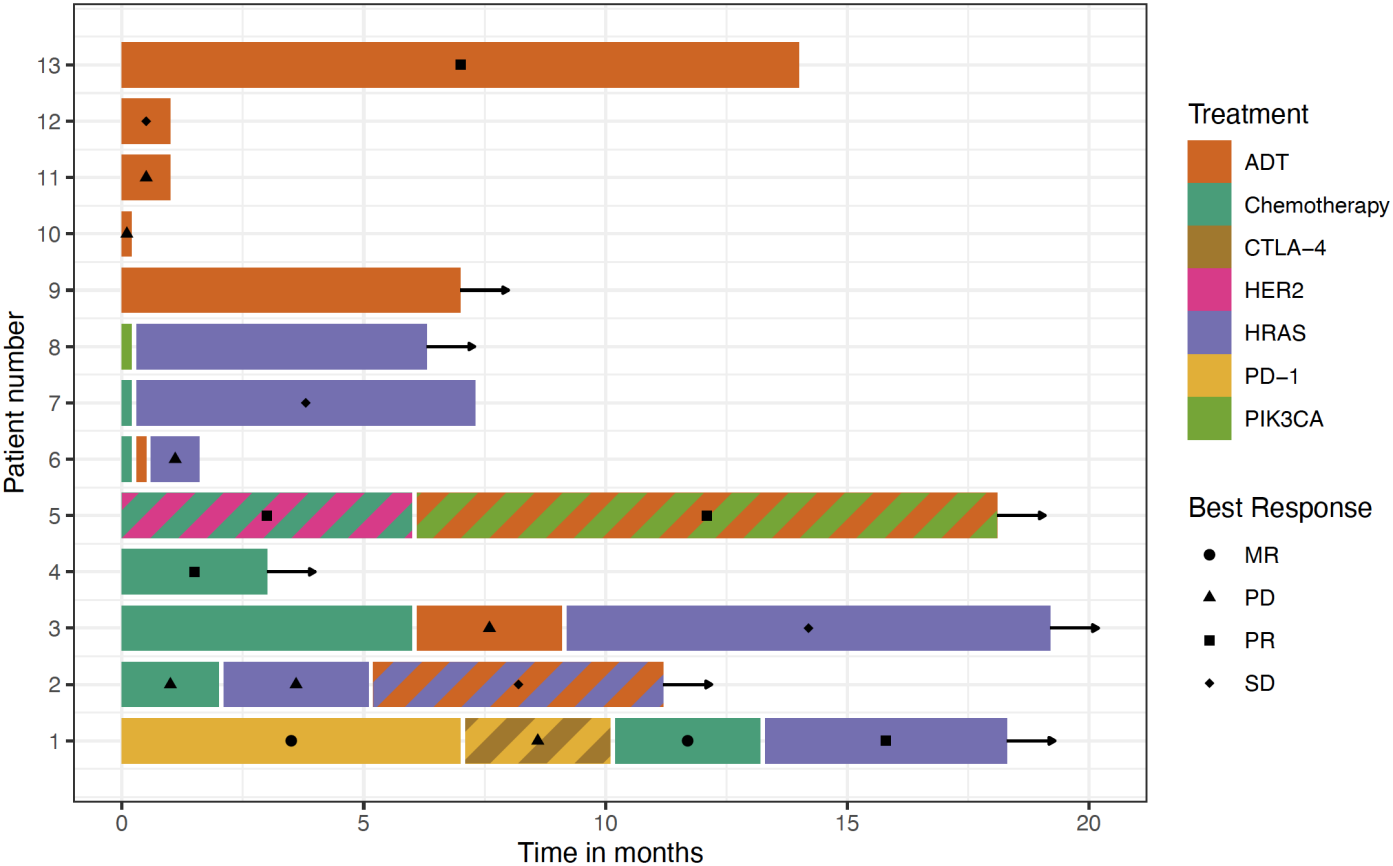
treatment sequences are depicted for the entire case series. No complete treatment sequences were available for patients 10-13. Duration of prior treatment was not provided for patients 6-8 (prior treatment indicated by colored bars). Color indicates the type of treatment; striped colors indicate combination therapy. Best responses are indicated. Arrows indicate ongoing therapy.

Combined analysis of 13 evaluable patients yielded information on various targeted systemic treatment strategies. Androgen deprivation therapy (ADT) was reported in 7 patients, *HRAS*-directed treatment in 6 patients, immune checkpoint inhibition in 1 patient and combinations of tipifarnib and ADT and alpelisib and ADT in 1 patient, each. Treatment data, including line of treatment, best response and progression-free survival (PFS) are provided, as available, in **Table 4** and **Figure 1**.

**Table 4:** Treatment and outcome data for the entire case series.

ID	Treatment #1	Best Response #1	PFS #1	Treatment #2	Best Response #2	PFS #2	Treatment #3	Best Response #3	PFS #3	Treatment #4	Best Response #4	PFS #4	OS
1	Pembrolizumab (off-label)	MR	7 m	Nivolumab/Ipilimumab (off-label)	PD	3 m	Carboplatin/Paclitaxel (off-label)	MR	3 m	Tipifarnib (compassionate use program)	PR	5 m+	18 m +
2	Carboplatin/Paclitaxel (off-label)	PD	2 m	Tipifarnib (compassionate use program)	PD	3 m	Tipifarnib/ADT (compassionate use program/off-label)	SD	6 m +				11 m +
3	Carboplatin/Paclitaxel (off-label)	n/a	6 m	ADT (Bicalutamid/Trenantone) (off-label)	PD	3 m	Tipifarnib (compassionate use program)	SD	10 m +				19 m +
4	Carboplatin/Paclitaxel (off-label)	PR	3 m +										3 m +
5	Carboplatin/Paclitaxel/Trastuzumab	PR	6 m	Alpelisib /ADT (Bicalutamid)	PR	12 m +							21 m +
6	Carboplatin	n/a	n/a	ADT	n/a	n/a	Tipifarnib	PD	1 m				n/a
7	Cisplatin	n/a	n/a	Tipifarnib	SD	7 m							n/a
8	Alpelisib	n/a	n/a	Tipifarnib	n/a	6 m +							n/a
9	ADT (Bicalutamid/Leuprolide)	n/a	7 m +										n/a
10	ADT (LHRH + bicalutamide) Treatment Sequence Unknown	PD	0 m										10 m
11	ADT (Bicalutamid) Treatment Sequence Unknown	PD	1 m										13 m
12	ADT (Bicalutamid) Treatment Sequence Unknown	SD	1 m										5 m
13	ADT (Bicalutamid) Treatment Sequence Unknown	PR	14 m										44 m

Best response and progression-free survival (PFS) data are indicated for each provided treatment line. + indicates ongoing therapy/survival. Isolated data for androgen deprivation therapy (ADT) but no complete treatment sequences were available for patients 10-13. No high-grade or unexpected toxicities were observed and no dose interruptions were necessary in patients 1-4. ADT, androgen deprivation therapy; ID, identification number; PD, progressive disease; PFS, progression-free survival; SD, stable disease; m, months; MR, mixed response, OS, overall survival; n/a, not available..

### 3.3 Androgen deprivation therapy

Seven patients were treated with androgen deprivation therapy alone (ADT). Among 6 patients with available data on the specific type of ADT, 3 received bicalutamide and a GnRH-analogue and 3 received bicalutamide alone. Best response was evaluable in 5 patients (1 PR, 1 SD, 3 PD). 6 patients had evaluable PFS (median PFS = 2 months) and 2 of them had PFS > 6 months.

### 3.4 *HRAS*-directed therapy

The farnesyltransferase inhibitor tipifarnib as a single agent was administered in 6 patients. Among 5 patients with available data, 1 PR, 2 SD and 2 PD were achieved as best responses. PFS data were available for 6 patients and PFS was more than 6 months in 3 patients.

### 3.5 Combination therapy

One patient received ADT (bicalutamid/GnRH-Analogue) in combination with tipifarnib after prior progression to tipifarnib after 3 months. This patient achieved stable disease for more than 6 months, which was ongoing at the time of data collection. Another patient achieved a partial response with the PI3K-inhibitor alpelisib in combination with ADT (bicalutamide) for more than 12 months (ongoing at time of publication).

### 3.6 Other treatment

Chemotherapy use with carboplatin/paclitaxel alone was reported in 4 patients. Among 3 patients with available data, 1 PR, 1 MR and 1 PD were reported. The use of alpelisib as monotherapy was only reported for one patient without information on treatment response. Immune checkpoint inhibition was also reported for one patient with a mixed response for 7 months. Following progression on the single-agent PD-1 inhibitor, this patient was treated with a combination of a PD-1 and a CTLA-4 inhibitor, which was followed by disease progression. One patient with concurrent *HER2* amplification received trastuzumab in combination with chemotherapy and achieved a partial response.

### 3.7 Toxicity

No major (common terminology criteria of adverse events, CTCAE grade 4 or higher) or unexpected toxicities were observed in the 4 patients identified from the MTB database and no dose reductions were required. In published data, a dose reduction because of toxicity was required in six patients (46%) receiving tipifarnib (4 because of cytopenia, 2 because of reversible renal failures), hypoglycemia requiring dose reduction was reported for alpelisib (9, 21). Toxicity data were not reported in the literature for patients receiving antiandrogen therapy in this cohort (22, 23). Available data from combined ADT in SGC reported no CTCAE grade 4/5 events and discontinuation of part of the combined ADT due to adverse events in 2 out of 36 patients (10).

## 4 Discussion

This is, to the best of our knowledge, the largest clinical case series of AR+, *PIK3CA*/*HRAS* co-mutated salivary duct carcinoma. The co-occurrence of these alterations has been described in previous analyses of this disease but was not associated with prognosis (19). The co-occurrence of these alterations poses a challenge for personalized therapy strategies. It is currently unclear, if response to targeted therapy is different in this subgroup. Despite the low number of patients, the heterogeneity of administered treatments in the cohort and the lack of data on mechanisms of secondary resistances, these results still hold important information because of the rarity of this disease. It should be noted that at least 2/13 patients in the cohort did not receive upfront palliative systemic therapy with ADT or chemotherapy (15, 16). This is likely caused by the lack of guidelines at the time of treatment initiation and a lack of data for the specific situation in AR+, *HRAS*/*PIK3CA* co-mutated tumors. Yet, despite available data and guidelines, current treatment strategies are not satisfactory and there is a lack of prospective clinical trials. The administration of experimental therapies, including immune checkpoint inhibition, will therefore likely remain a reality in these tumors, thus making sharing of real-world data essential.

HER2 overexpression or amplification is a well-defined therapeutic target in SDC (11). HER2 positivity has been found to be mutually exclusive with the AR+, *HRAS*/*PIK3CA* co-mutated subgroup (19). In this cohort, concurrent HER2 expression or amplification was reported in 6 patients with mostly low to moderate staining intensity. One patient was reported to harbor a concurrent *HER2* amplification and received trastuzumab in combination with chemotherapy, achieving a partial response. These results suggest, that HER2-positivity is not entirely mutually exclusive with the here described subgroup and HER2-directed treatment might be an additional option in some of the patients. In addition to HER2-directed antibodies, efficacy of HER2-directed antibody drug conjugates was shown in salivary gland cancer (24).

In the group of patients treated with ADT, a clinical benefit was observed in about a third of the evaluable patients, which is less than the clinical benefit rate of about 75% in previously reported results (10). However, clinical benefit was found in some patients, thus providing evidence of activity and the low number of patients does not allow further conclusions. Furthermore, previous work did not show an impact of oncogenic drivers, including *HRAS* and *PIK3CA* mutations, on the efficacy of ADT but studies on larger cohorts are warranted (10). Therefore, these data do not provide evidence against the use of ADT in the AR+, *HRAS*/*PIK3CA* co-mutated subgroup. Importantly, no data currently exist for the use of abiraterone in castration-resistant AR+, *HRAS*/*PIK3CA* co-mutated SDC. Abiraterone is active as a second line ADT in AR+ salivary gland cancer and might represent an additional treatment strategy for the here reported subgroup (13). Additional data on limited activity with enzalutamide were reported previously (14). In this phase 2 trial, tumor regressions were also noted among patients with prior ADT (14).

In patients receiving *HRAS*-directed therapy, 1 objective response and clinically meaningful disease stabilizations in about half of the patients was reported. These results are similar to previously reported results in SGC (9). In the same trial, co-occuring *PIK3CA*-alterations or the type of *HRAS* mutation (more common Q61 or less common G13) did also not seem to impact treatment efficacy (9). Again, these data further support the investigation of tipifarnib in the here reported disease subgroup.

Two patients received combination therapy after prior progression of disease. One patient achieved disease stabilization with tipifarnib and ADT after prior progression with tipifarnib monotherapy. In this patient, tipifarnib treatment was continued because of low toxicity and improvement in local symptoms. However, disease stabilization might be mediated by ADT alone. Another patient achieved a partial response with the PI3K-inhibitor alpelisib and ADT. The impact of the individual drugs in this combination therapy can also not be assessed. Further investigation of combination therapies is warranted.

A single agent PI3K-inhibitor was only used in 1 patient and no response data were available. The published results from the NCI-MATCH subprotocol Z1F of Copanlisib in *PIK3CA*-mutated cancer did show activity of the drug in several cancer types, but no SDC were enrolled (25). Additional research is needed to establish the activity of single-agent PI3K-inhibitors in SDC.

The activity of immune checkpoint inhibition also remains to be determined. One patient in the reported cohort achieved a mixed response with single-agent PD-1 blockade for 7 months, followed by disease progression. The same patient then experienced disease progression with combined PD-1 and CTLA-4 inhibition. Immune checkpoint inhibition was administered in this patient because of high PD-L1 expression and the co-occurrence of driver alterations, potentially complicating single-agent targeted treatment. These results suggest, that immune checkpoint inhibitors might be an additional treatment option in some patients. An analysis of 109 patients with advanced SGC in the Keynote-158 study showed an overall response rate of 4.6% in the overall population and 10.7% (n=3/28) in the PD-L1 positive population (26). Only 3 patients in this trial were found to be TMB-high, among which 1 had an objective reponse (26). PD-L1 expression or a high TMB > 10 mut/Mb was reported in 2 patients in the here reported cohort, accordingly.

For untargeted therapies, best data currently exist for carboplatin/paclitaxel use, which is also supported by previous analyses and yields clinically meaningful benefit (27).

In summary, the here provided data show multiple targeted treatment strategies for patients with AR+, *HRAS*/*PIK3CA* co-mutated SDC. Best available evidence, expected toxicities and patient factors need to be considered for a choice of treatment in this rare subgroup. These results support comprehensive molecular profiling of SDC. Additional molecular analyses might help with further establishing active signaling pathways for treatment stratification. Further research is required to establish optimal treatment combinations and sequences, which is a challenge in this rare disease.

## Data availability statement

The original contributions presented in the study are included in the article/**Supplementary Material**. Further inquiries can be directed to the corresponding author.

## Ethics statement

The studies involving human participants were reviewed and approved by Charité - Universitätsmedizin Berlin. Written informed consent for participation was not required for this study in accordance with the national legislation and the institutional requirements.

## Author contributions

DR, SS, PS and KK provided patient data, DR performed a systematic review of the literature, DR, EZ, BE, DB, EB, UK (8^th^ Author), UK (9^th^ Author) and KK analyzed data. DR, UK (9^th^ Author) and KK wrote the manuscript. All authors contributed to the article and approved the submitted version.

## Supplementary material

Supplementary Figure 1 Consort diagram showing the identification of patients for the final cohort. MTB, molecular tumor board; HNC, head and neck cancer; SGC, salivary gland cancer; SDC, salivary duct carcinoma.
