# Supplementary figures and images for: Targeted treatment in a case series of AR+, HRAS/PIK3CA co-mutated salivary duct carcinoma
Source: Front Oncol. 2023 Jun 20;13:1107134. doi: 10.3389/fonc.2023.1107134 (PMC10325704; doi:10.3389/fonc.2023.1107134)

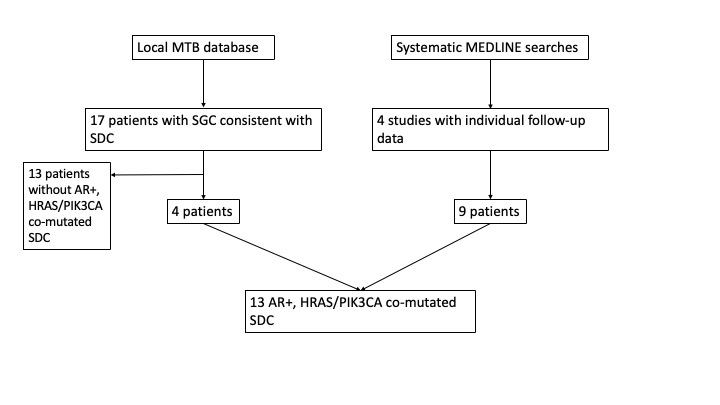

Supplement: Supplementary Figure 1 — Consort diagram showing the identification of patients for the final cohort. MTB, molecular tumor board; HNC, head and neck cancer; SGC, salivary gland cancer; SDC, salivary duct carcinoma. [file Image_1.jpeg]
